# Complete mitochondrial genome sequence of *Fusarium tricinctum*

**DOI:** 10.1080/23802359.2020.1773335

**Published:** 2020-06-08

**Authors:** Kuizhong Xie, Yun Yue, Xinyuan Hu

**Affiliations:** a Potato Research Institute, Gansu Academy of Agricultural Sciences, Lanzhou, Gansu, People’s Republic of China; b Gansu General Station of Agro-technology Extension, Lanzhou, Gansu, People’s Republic of China

**Keywords:** *Fusarium tricinctum*, mitochondrial genome, Fusarium commune

## Abstract

The complete mitochondrial genome of *Fusarium tricinctum* was sequenced. The circular molecule is a total length of 46,314 bp, and the base composition of the mitogenome is as follows: A (33.6%), T (33.1%), C (15.1%), and G (18.2%). The mitogenome contains 19 protein-coding genes, 2 ribosomal RNA (rRNA), and 27 transfer RNA (tRNA) genes. The mitogenome analysis of *F. tricinctum* provides a molecular basis for further studies on molecular systematics and evolutionary dynamics.

*Fusarium* is a large genus of filamentous fungi, which is widely distributed in soil and associated with plants (Nelson et al. 1994). Most species in this genus are harmless saprobes and also include a number of economically important plant pathogenic species (Moretti 2009). *Fusarium tricinctum* strain KGS17 (GenBank accession number MN365076) was isolated from potato root that was rotted in Gansu, northwest China (35°26.346′N, 104°50.991′E) and identified based on translation elongation factor 1a gene and internal transcribed spacer (White et al. 1990; Samson et al. 2004). The voucher specimen (No. KGS17) was deposited at the Gansu Provincial Key Lab of Aridland Crop Science, Lanzhou, Gansu, China. *Fusarium tricinctum* was stored in Gansu Academy of Agricultural Sciences. The total genomic DNA mycelia obtained was extracted using Fungal DNA Kit D3390-02 (Omega Bio-Tek, Norcross, GA) according to the manufacturer’s instructions and was stored in the sequencing company (Xuan Chen Biological Technology Co., Ltd. Shaanxi, China). Purified DNA was used to construct the sequencing libraries following the instructions of NEBNext® Ultra^TM^ II DNA Library Prep Kit (NEB, Beijing, China). Whole genome sequencing was performed using the Illumina novaseq6000 Platform (Illumina, San Diego, CA). Multiple steps were used for quality control and *de novo* assembly of the mitogenome (Bi 2017). Adapters and low-quality reads were removed using the NGS QC Toolkit (Patel and Jain 2012).

The obtained clean reads were screened out by Bowtie 2 (Langmead and Salzberg 2012) with the help of homologous reference sequence (Fusarium commune: LT906348), and then assembled as implemented by NOVOPlasty v3.8.3 (Dierckxsens et al. 2017). Genome annotation is mainly carried out by comparing the mitochondrial genome (see phylogenetic tree for details) of the same genus and relative species of GenBank. Geneious R11 software (https://www.genetic.com) was used to make mitochondrial genome map. The length of *F. tricinctum* mitogenome is 46,314 bp, and its size, structure, and gene content are similar to those of *Fusarium*. This mitogenome was submitted to the GenBank database under accession no. MT269798. The circular mitogenome contains 19 protein-coding genes, 2 ribosomal RNA (rRNA), and 27 transfer RNA(tRNA) genes (Figure 1). The base composition of the genome is as follows: A (33.6%), T (33.1%), C (15.1%), and G (18.2%).

**Figure 1.**
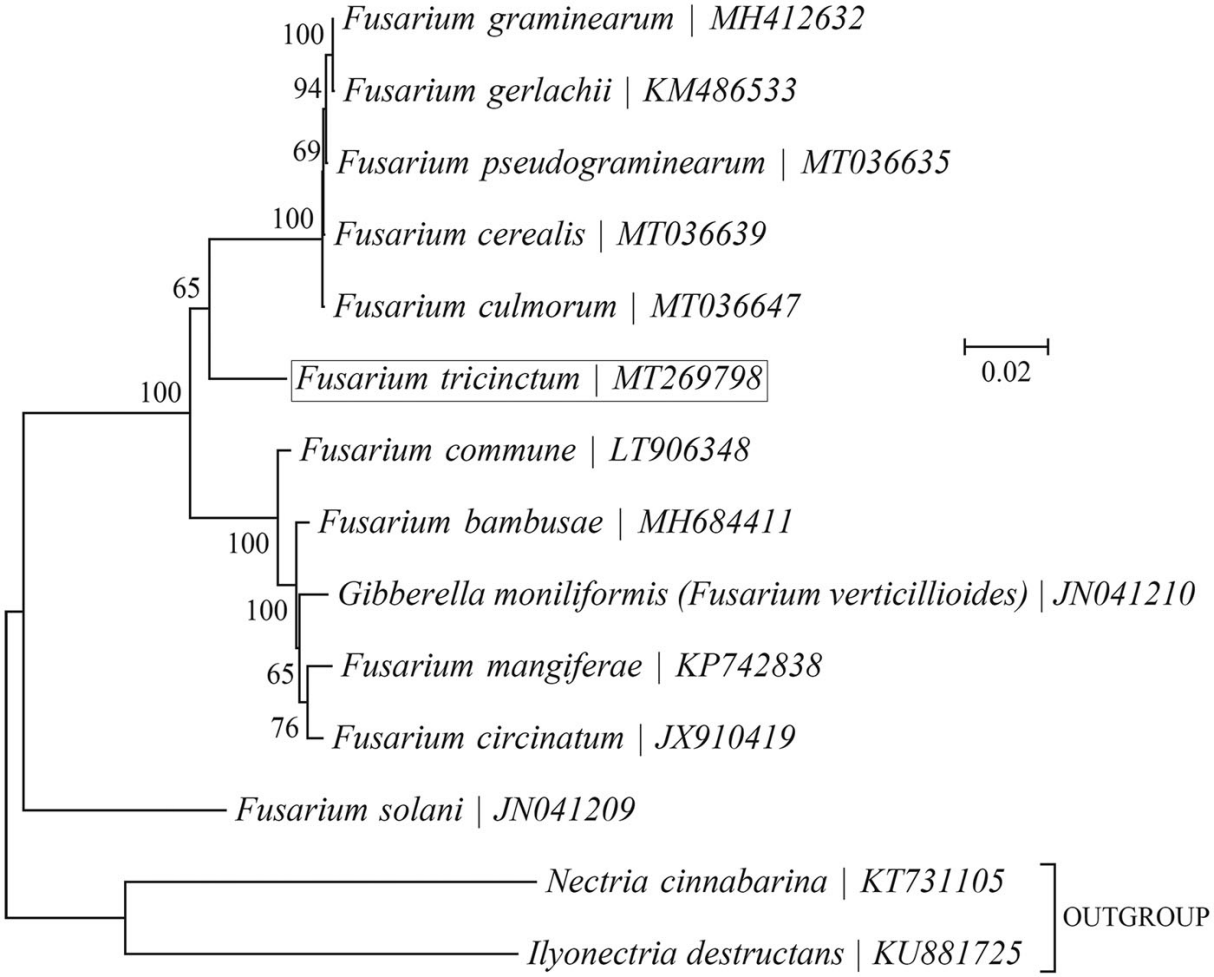
Phylogenetic relationships among 13 *Fusarium* mitogenomes. This tree was drawn with potato *Fusarium tricinctum* as an outgroup. The length of branch represents the divergence distance.

To validate the phylogenetic position of *F. tricinctum*, the genome-wide alignment of *Fusarium* mitogenomes was constructed by Geneious R11 software (https://www.genetic.com). Genes with good comparison effect were selected and then connected into a single super gene. It was exported to topali v2.5 software (Milne et al. 2009) to build the phylogenetic tree (Figure 1).

As shown in Figure 1, *Fusarium cerealis* (MT036639) and *Fusarium culmorum* (MT036647) are determined as sisters of *Fusarium tricinctum* with strong support. High bootstrap and posterior probability values show that presented relations are stable. The mitochondrial genome of *Fusarium tricinctum* will contribute to the understanding of phylogeny.

## Data Availability

The data that support the findings of this study are openly available in NCBI GenBank database at (https://www.ncbi.nlm.nih.gov/nuccore/) with the accession number is MT269798, which permits unrestricted use, distribution, and reproduction in any medium, provided the original work is properly cited.
